# Contraceptive dynamics among women with disabilities in low- and middle-income countries: a scoping review protocol

**DOI:** 10.1186/s13643-023-02214-4

**Published:** 2023-03-14

**Authors:** Getalem Aychew Beyene, Solomon Mekonnen Abebe, Gedefaw Abeje Fekadu, Achenef Asmamaw Muche, Bisrat Misganaw Geremew

**Affiliations:** 1 Institute of Public Health, College of Medicine and Health Sciences, University of Gondar, Gondar, Ethiopia; 2 Plan International Ethiopia, Bahir Dar, Ethiopia; 3 Department of Reproductive Health and Population Studies, College of Medicine and Health Sciences, Bahir Dar University, Bahir Dar, Ethiopia

**Keywords:** Women, Disability, Contraceptive Dynamics, Low- and middle-income countries

## Abstract

**Introduction:**

Contraceptive dynamics is the use of contraception, unmet need, discontinuation, and/or switching of contraception. Women with disabilities (WWDs) in low- and middle-income countries (LMICs) face a common problem: a low prevalence of contraceptive usage and a high unmet need. Even though certain studies have been conducted in high-income countries, research is scarce on the degree of contraceptive method mix, unmet needs, contraception discontinuation, and switching among WWDs in LMICs. As a result, the scoping review’s goal is to investigate, map available evidence, and identify knowledge gaps on contraceptive dynamics within LMICs WWDs.

**Methods:**

The scoping review is guided by the six-stage Arksey and O’Malley methodology framework. Published articles will be retrieved from databases such as PubMed (MEDLINE), the Cochrane Library, Cumulative Index to Nursing and Allied Health Literature (CINAHL), and Global Health. Grey literature databases will be searched using electronic search engines such as Google Scholar, Google, OpenGrey, and Worldcat. In addition, a manual search of reference lists from recognized studies will be conducted, as well as a hand search of the literature. Any type of study design (e.g., randomized controlled trials, quasi-experimental studies, prospective and retrospective cohort studies, case-control or nested case-control studies, qualitative, cross-sectional studies) will be included in this scoping review. There will be no restrictions on publication year. Two independent reviewers will screen relevant publications, and data will be charted accordingly. The Preferred Reporting Items for Systematic Review and Meta-Analyses Extension for Scoping Reviews (PRISMA-ScR) checklist and reporting guidelines will be used to report all parts of the protocol and scoping review.

**Discussion:**

When compared to non-disabled women, WWDs had a lower prevalence of contraceptive usage and a higher unmet need in LMICs. Despite these facts, they are the most marginalized people on the planet. This is, therefore, critical to map available evidence and identify knowledge gaps on contraceptive dynamics. As a result, the findings of this scoping review will be significant in terms of the contraceptive dynamic among WWDs in LMICs.

**Systematic review registration:**

Open Science Framework (OSF), with registration number; DOI/10.17605/OSF.IO/XCKPT.

**Supplementary Information:**

The online version contains supplementary material available at 10.1186/s13643-023-02214-4.

## Introduction

The International Classification of Functioning Disability and Health (ICF) describes a disability as any impairment of physical structures and functions and restrictions on activity and participation [1]. Around 15% of the world’s population has some form of disability, with many of them being disproportionately impacted by poverty, according to 2011 research from the World Health Organization (WHO) and the World Bank [2]. Over 80% of the population in LMICs are people with disabilities (PWDs), of whom 60 million live in Africa and 7.7 million in Ethiopia [3, 4]. PWDs made up 12.7%, 14%, and 16.8% of the population, respectively, in Ethiopia’s Oromia, Amhara, and South Nations and Nationalities of Peoples (SNNPs) regions [5].

PWDs are frequently viewed as one of the most excluded and marginalized groups in society [6]. Due to their greater disadvantage in many developing countries, women with disabilities have much worse conditions than men with impairments [7, 8].

Contraceptive dynamics is the use of contraception, unmet need, discontinuation, and/or switching of contraception [9]. Access to and usage of contraception are essential for achieving the recently unveiled Sustainable Development Goals (SDGs) [10]. To protect and promote PWDs’ human rights, advance the global development agenda, and create a society that is truly inclusive, it is imperative to pay attention to contraception services and PWDs’ SRH requirements [11]. Contraceptive use for people with disabilities (WWDs) is a concern for all bodies due to the marginalization and exclusion of this population from active engagement in society compared to their non-disabled counterparts [12].

Due to their limited access to family planning (FP) clinics, information, and services, disabled women do not make advantage of the services offered by FP clinics [13]. Contraception is undoubtedly a critical unmet need, even though the full picture of SRH challenges for PWDs is not quite understood [14].

Contraception can lessen chronic hunger and poverty by preventing unplanned pregnancies and lowering fertility rates. It can also increase access to high-quality education, raise gender equality, improve maternal health, and minimize childhood mortality. It can also help to ensure the long-term use of natural resources, as well as slow down the rate of climate change and lessen the frequency and severity of conflicts around the world by minimizing the amount of resource competition brought on by population expansion [15, 16].

Because PWDs are viewed as asexual and unlikely to get married or have children, disability is one of the biggest obstacles to using reproductive health (RH) services, particularly family planning (FP), in sub-Saharan Africa. When it comes to the number of women who desire to space out, limit, or stop having children, WWDs have a significant unmet need for FP. The few local area studies on the RH of WWDs in LMICs show that WWDs are neglected and excluded from contraceptive availability and services in comparison to normal women [17, 18]. Although detailed studies of specific concerns such as handicaps and HIV have been undertaken in some areas of LMICs, there has been little research on the contraception practices of WWDs in these contexts. Although comprehensive studies of particular issues, such as handicaps and HIV, have been conducted in some regions of LMICs, there has not been much research on WWDs’ contraceptive practices in these settings [19, 20].

A thorough examination of the evidence of contraceptive dynamics among WWDs, as well as the amount of application and effectiveness of contraceptive services in LMICs, is critical in determining the emphasis of future research on the disabled population. Scanning the corpus of literature on a given issue, summarizing and distributing research findings, identifying research gaps, and offering recommendations for future study are all essential purposes of scoping reviews. This scoping review aims to improve our understanding of contraceptive use, unmet needs, switching, and discontinuation among WWDs in LMICs. The findings of this study will allow researchers to assess the scope and diversity of research on contraceptive dynamics among WWDs in LMICs.

### Objectives

The main objective of the proposed scoping review is to identify, explore and map the available evidence on contraceptive use, unmet need, switching, discontinuation, and current contraceptive care models among WWDs in LMICs. It is anticipated that the results of a scoping review will inform governments and policymakers of the FP services required by WWDs in LMICs and identify gaps for further research.

## Methodology

### Scoping review

This protocol is for a scoping review of literature reporting on contraceptive dynamics among WWDs in LMICs. The procedures specified by Arksey and O’Malley’s scoping review methodology, as well as Levac et al.’s and Peters et al.’s scoping methodology enhancement recommendations [21–23], will be followed in this scoping review. Thus, the following six steps will be followed in this scoping review: (1) identifying the research question; (2) identifying relevant studies; (3) selecting studies; (4) charting data; (5) collating, summarizing, and reporting the results; and (6) consulting with relevant stakeholders. A quality appraisal will not be done as this review aims to map all research activities in this field.

#### Stage one: identifying the research question

The main research question is “What is known about the contraceptive dynamics and models of contraceptive care among WWDs in LMICs?” This question would allow us to review and capture the full scope of existing literature while also allowing for the addition or modification of guiding research topics in an iterative manner.

The following sub-questions will be addressed:

1. What forms of contraceptive dynamics on WWDs have been studied in LMICs so far?
2. Where were contraceptive dynamics studies conducted in LMICs?
3. What types of disabilities have been included in the studies?
4. What is the prevalence of contraceptive use among WWDs in LMICs, as well as unmet needs, contraceptive discontinuation, and switching of contraceptive use?
5. What are the challenges faced by WWDs to access contraceptive services?

#### Eligibility criteria

The Population-Concept-Context (PCC) framework, as recommended by updated methodological guidance for the scoping review by Peter et al. [24] the Joanna Briggs Institute for scoping reviews [25], will be utilized in the study to determine the eligibility of the research question (see Table 1). This is a more flexible alternative to the PICO (Population, Intervention, Comparator, and Outcome) framework recommended for systematic reviews.

**Table 1:** PCC framework

Population	Reproductive-age women with disabilities (15–49 years of age)
Concept	Contraceptive dynamics. This includes contraceptive use, unmet need, switching, and discontinuation Disability defined by WHO ICF as disability is any impairment in body functions and structures, limitations in activity, and restriction in participation.
Context	Low- and middle-income countries

For studies to be included, they must meet the following criteria:

- Focus on WWDs of reproductive age groups
- Studies focus on contraceptive use, unmet need, switching and discontinuation, and models of contraceptive care
- Include participants from LMICs
- No time of restriction
- Any type of study design (e.g., randomized controlled trials, quasi-experimental studies, prospective and retrospective cohort studies, case-control or nested case-control studies, qualitative, and cross-sectional studies)
- Published in the English language

Studies will be excluded if they have any of the following characteristics.

- Studies focus on women without disabilities
- Studies do not focus on contraceptive use, unmet need, switching, and discontinuation and models of contraceptive care
- Studies that do not include participants or studies from LMICs
- Studies published other than in the English language
- Studies where the full-text article could not be obtained

#### Stage two: identifying relevant studies

##### Search strategy and information sources

The following databases will be searched for eligible studies: PubMed (MEDLINE), CINAHL, Cochrane databases, and Global Health will be searched for articles that are not indexed in PubMed. We will additionally explore a range of grey literature sources to ensure that other important information is gathered. We will look through grey literature resources (including Google Scholar, Google, OpenGrey, and WorldCat) to find research, reports, and conference abstracts that are relevant to this review.

In addition, we will manually search the reference lists of all relevant material. A library will be created for this review using EndNote x9 referencing software. The results of the search will be downloaded into a citation manager and imported into an EndNote library for further inspection and duplication detection.

We used different synonyms of LMICs and the World Bank Country and Lending Groups June 2020 fiscal year list of LMICs (https://blogs.worldbank.org/opendata/newcountry-classifications-income-level-2019-2020). The Literature search will be conducted by experienced research team members. GAF and AAM are an expert and trainers of literature searches and systematic reviews. GAB has completed a 5-day intensive training on literature searches and systematic reviews. Moreover, we will be using established methods to ensure the quality of the literature search, screening, and information charting. The search strategy will be piloted to check the appropriateness of keywords and databases. The electronic database search will be recorded in a table. A draft is provided in Table 2.

**Table 2:** Electronic database searches

Electronic database	Key words to be searched
PubMed	“women” [All Fields] OR “women” [MeSH Terms] OR "reproductive age” [All Fields] OR “15-49 years” [All Fields] **AND** “Disabili*” [All Fields] OR “disabili*” [MeSH Terms] OR “impairmen*” [All Fields] OR "physical disabilit*" [All Fields] OR "visual impairmen*"[All Fields] OR “visual loss” [All Fields] OR “blind” [All Fields] OR blind [MeSH Terms] OR “hearing loss”[All Fields] OR "hearing loss" [MeSH Terms] OR “hearing impairmen*” [All Fields] OR “deaf” [All Fields] OR "intellectual disabili*" [All Fields] OR “intellectual disabili*” [MeSH Terms] OR "sensory disabili*" [All fields] **AND** “Birth control” [All Fields] OR "family planning services" [All Fields] OR "family planning services" [MeSH Terms] OR “contraception behavior” [MeSH Terms] OR “contraception/psychology” [MeSH Terms] OR “contraception/utilization” [All Fields] OR “family plannin*” [All Fields] OR “contracepti*” [All Fields] OR “contracepti*” [MeSH Terms] OR “contraceptive agen*” [All Fields] OR “contraceptive agen*” [MeSH Terms] OR “contraceptive methods” [All Fields] OR “contraceptive device*” [MeSH Terms] OR “contraceptive devic*” [All Fields] OR “planned pregnanc*” [All Fields] OR “birth prevention*” [All Fields] OR “prevent pregnanc*” [All Fields] OR “birth interva*” [MeSH Terms] OR “birth interva**” [All Fields] OR “birth spacing” [All Fields] OR “pregnancy interval” [All Fields] OR “pregnancy spacing” [All Fields] **AND** “Dynami*” [All Fields] OR “dynami*” [MeSH Terms] OR “Utilizatio*” [All Fields] OR “utilizatio*” [MeSH Terms] OR “use” [All Fields] OR “practic*” [MeSH Terms] OR “practic*” [All Fields] OR "unmet nee*" [All Fields] OR “discontinuatio*”[All Fields] OR "dis-continuatio*" [All Fields] OR “switchin*” [All Fields] OR “chang*” [All Fields] OR “chang*” [MeSH Terms] **AND** “middle income countr*” [All Fields] OR “low income countr*” [All Fields] OR “developing countr*” [All Fields] OR “resource-limited countries” [All Fields]

#### Stage three: study selection

*The review process will consist of two levels of screening*: (1) a title and abstract review and (2) a full-text review

**Fig. 1:**
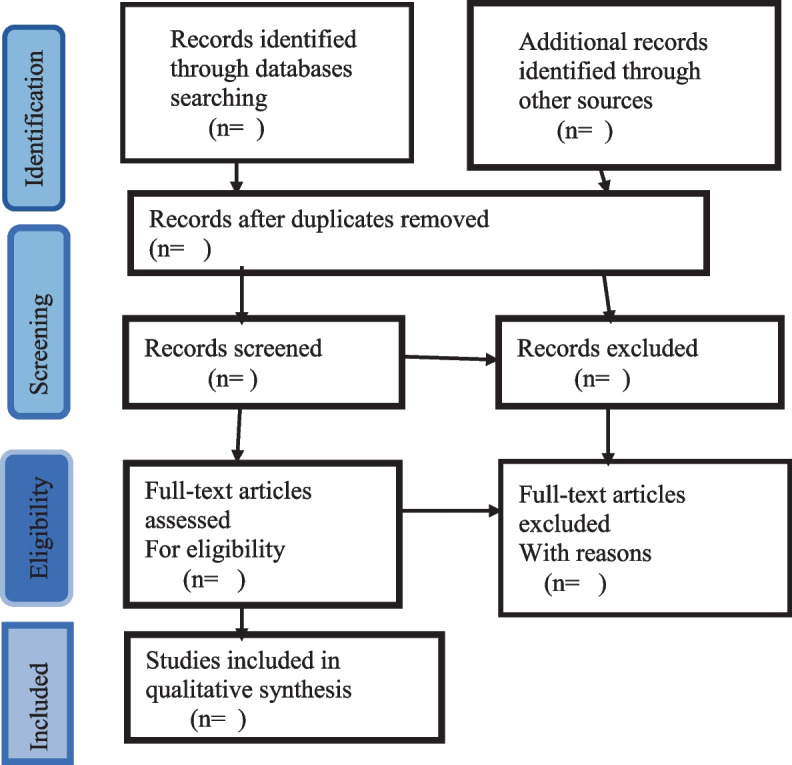
PRISMA-ScR flow diagram of literatures to be searched

For the first level screening, two researchers will independently screen each retrieved citation’s title and abstract for inclusion using a set of minimum inclusion criteria. Before starting the abstract review, the criteria will be tested on a sample of abstracts to make sure they are strong enough to capture any papers that might pertain to contraceptive dynamics. The full-text review will contain articles that are deemed relevant by either or both reviewers. In the second step, the two investigators will then independently assess the full-text articles to determine if they meet the inclusion/exclusion criteria. Any discordant full-text publications will be re-evaluated, and any remaining issues concerning research eligibility will be resolved by discussion at the full-text review stage. At this point, a third reviewer may be called to help resolve any disagreements. The selection process will follow the recommendations in the Preferred Reporting Items for Systematic Reviews and Meta-Analyses Extension for Scoping Reviews (PRISMA-ScR) chart [26], shown in Fig. 1.

Reviewers will get together at the beginning, midpoint, and end of the abstract review process to discuss any problems or ambiguities with study selection and to go back and adjust the search strategy as necessary. Throughout the duration of the evaluation process, the number of studies that were included and excluded will be recorded. A calibration experiment will be conducted on 20 randomly chosen papers before screening and charting to ensure team consensus. A statistical measure will be used to evaluate the internal validity and inter-rater reliability of the selection method used in our study. It has been determined that a threshold of 80% agreement between coders is acceptable [27]. The evaluation procedure will be thoroughly documented so that the study can be reproduced by others.

#### Stage four: charting the data

The study team created a data-collecting instrument to retrieve data from the included studies for both the contraceptive dynamic and the model of contraception care. The team built the data charting form together and decided on the variables and level of detail of the data to be extracted. The study team will pilot the tool before the start of the review to ensure that it appropriately captures the information.

The data will be abstracted by two independent reviewers, and the abstracted data will be compared. The handling of any discrepancies will guarantee that the reviewers are on the same page. According to the recommendations made by Arksey and O’Malley and Peters et al. for the scoping review, the data mining process will include descriptive analytical techniques that transparently summarize and synthesize information. Based on the Joanna Briggs Institute (JBI) scoping review manual [28] and Peter et al.’s scoping review guidance [23], a data charting table (Table 3) will be used to record characteristics of the included studies as well as critical information pertinent to the review question. In the conduct and reporting of this scoping review, we will use EndNote to organize and code references [29].

**Table 3:** Data charting form

Author and date	
Country of origin	
Aim or purpose of the study	
Study setting	
Study population (type of disability)	
Sampling method	
Study design	
Data collection methods	
Data analysis	
Key findings	Contraceptive dynamics; contraceptive use, unmet need, switching and discontinuation of contraceptive use

#### Stage five: collating, summarizing, and reporting the results

We will present stage five in three discrete parts, as recommended by Levac et al.: assessing the data, reporting results, and applying meaning to the results [22]. The data will be summarized and reported in a way that maps the breadth of extant literature in the field of contraceptive dynamics and its model of contraception care in LMICs. We will map the concepts that underpin contraceptive dynamic research, as well as the types and quality of evidence available in LMICs. Furthermore, the available evidence on contraception dynamics and care models in LMICs will be mapped and described in detail. We will narrate the implications of findings within the larger framework for research, policy, and practice to make this scoping review more usable.

Finally, we will give an overview of the research field and where it is at right now, as well as the gaps that exist. Over the course of 3 months, the review team plans to conduct preliminary searches and complete literature searches, screening, and data charting. The results will then be gathered, summarized, and reported.

#### Stage six: consulting with relevant stakeholders

Consultation, according to Arksey and O’Malley, enhances methodological rigor. Once the early findings from stage five have been produced, seeking stakeholders’ opinions (policymakers, practitioners, and researchers in Ethiopia) and their thoughts on applying the results to the scoping study would be considered.

Since it is not a typical requirement of scoping reviews, we will not perform a quality assessment of the papers to be evaluated [30, 31]. However, we will use the parts of the Peer Review of Electronic Search Strategies (PRESS EBC Elements) [32] to improve the accuracy and completeness of the evidence-based search.

## Discussion

PWDs are among the most socially and economically disadvantaged groups in the world [6]. Due to their greater disadvantage in many developing countries, women with disabilities have much worse conditions than men with impairments [7, 8]. People with disabilities do not use the services provided by family planning (FP) clinics because access to them is limited and information and services are few [7]. Furthermore, the deleterious effects of accessing contraception services are numerous. For instance, it could lead to unintended pregnancy, abortion, adverse pregnancy outcomes, and high maternal morbidity and mortality.

WWDs showed a lower prevalence of contraceptive use and a higher unmet need as compared to non-disabled women in low- and middle-income countries. In WWDs, the prevalence of contraception ranged from 13 to 31.1%, with 24.3% of unmet needs [33–35]. This indicates a pervasive issue that could compromise the United Nations (UN) General Assembly Convention article 25, which guarantees PWDs access to SRH services and make the situation difficult to address. Despite these facts, they are the most marginalized people on the planet.

In order to reduce high unmet needs and contraception failure, it is essential to design a contraception model of care to support access to contraception. It is crucial to map the current research and pinpoint knowledge gaps regarding the dynamics of contraception among WWDs. The results of this scoping review will therefore be important for understanding contraception dynamics among WWDs in LMICs.

To our knowledge, this is the first scoping review that will provide a complete overview and insight on contraceptive dynamics among WWDs in LMICs. This review’s strength will be its ability to clearly identify existing knowledge gaps on contraceptive dynamics while employing a transparent and repeatable procedure. The review’s limitation is that only English literature will reflect a portion of the study done in LMICs. It will, however, make every effort to present a clear picture of contraceptive dynamics in LMICs, regardless of publication year or status.

## Dissemination and ethics

This will be the first scoping review of its sort and will describe the various types of evidence in contraceptive dynamics, delineate key concepts in the literature, provide good insight into contraception care models, and assess evidence gaps in LMICs.

Researchers will be able to identify any knowledge gaps using the findings. The findings of this scoping study will be applied to direct further investigations into the contraceptive dynamics in Ethiopia and other LMICs. No ethical approval is needed for this project because the scoping review approach uses publicly available materials to review and collect data [36].

The suggested scoping review is viable, attainable, and timely, in our opinion. At local, national, and worldwide conferences, we will develop presentations to distribute findings to key stakeholder and end-user groups. Our findings will also be published in a peer-reviewed journal. We reviewed the International Prospective Register of Systematic Reviews to see if any review protocols on the same issue had been registered (PROSPERO). This scoping review protocol was registered in the Open Science Framework (OSF) database @osf.io/7btvn with the registration number DOI/10.17605/OSF.IO/XCKPT because PROSPERO is not presently accepting scoping review protocols for registration.

## Supplementary Information

**Additional file 1.** Search results from PubMed.

**Additional file 2.** PRISMA.

## Data Availability

Not applicable.

## Abbreviations

FP: Family planning
LMICs: Low- and middle-income countries
MeSH: Medical Subject Headings
PRISMA-ScR: Preferred Reporting Items for Systematic Review and Meta-Analyses Extension for Scoping Reviews
PWDs: People with disabilities
RH: Reproductive health
SRH: Sexual reproductive health
WHO: World Health Organization
WWDs: Women with disabilities
