# Supplementary material for: Contraceptive dynamics among women with disabilities in low- and middle-income countries: a scoping review protocol
Source: Syst Rev. 2023 Mar 14;12:40. doi: 10.1186/s13643-023-02214-4 (PMC10012547; doi:10.1186/s13643-023-02214-4)
Supplement: Supplementary file 1 — Additional file 1. Search results from PubMed. [file 13643_2023_2214_MOESM1_ESM.docx]

| Search number | Query | Sort By | Filters | Search Details | Results |
| --- | --- | --- | --- | --- | --- |
| 17 | (((("women" [All Fields] OR "women" [MeSH Terms] OR "reproductive age" [All Fields] OR "15-49 years" [All Fields]) AND ("Disabili*" [All Fields] OR "disabili*" [MeSH Terms] OR "impairmen*" [All Fields] OR "physical disabilit*" [All Fields] OR "visual impairmen*"[All Fields] OR "visual loss" [All Fields] OR "blind" [All Fields] OR blind [MeSH Terms] OR "hearing loss"[All Fields] OR "hearing loss" [MeSH Terms] OR "hearing impairmen*" [All Fields] OR "deaf" [All Fields] OR "intellectual disabili*" [All Fields] OR "intellectual disabili*" [MeSH Terms] OR "sensory disabili*" [All fields])) AND ("Birth control" [All Fields] OR "family planning services" [All Fields] OR "family planning services" [MeSH Terms] OR "contraception behavior" [MeSH Terms] OR "contraception/psychology" [MeSH Terms] OR "contraception/utilization" [All Fields] OR "family plannin*" [All Fields] OR "contracepti*" [All Fields] OR "contracepti*" [MeSH Terms] OR "contraceptive agen*" [All Fields] OR "contraceptive agen*" [MeSH Terms] OR "contraceptive methods" [All Fields] OR "contraceptive device*" [MeSH Terms] OR "contraceptive devic*" [All Fields] OR "planned pregnanc*" [All Fields] OR "birth prevention*" [All Fields] OR "prevent pregnanc*" [All Fields] OR "birth interva*" [MeSH Terms] OR "birth interva**" [All Fields] OR "birth spacing" [All Fields] OR "pregnancy interval" [All Fields] OR "pregnancy spacing" [All Fields])) AND ("Dynami*" [All Fields] OR "dynami*" [MeSH Terms] OR "Utilizatio*" [All Fields] OR "utilizatio*" [MeSH Terms] OR "use" [All Fields] OR "practic*" [MeSH Terms] OR "practic*" [All Fields] OR "unmet nee*" [All Fields] OR "discontinuatio*"[All Fields] OR "dis-continuatio*" [All Fields] OR "switchin*" [All Fields] OR "chang*" [All Fields] OR "chang*" [MeSH Terms])) AND ("middle income countr*" [All Fields] OR "low income countr*" [All Fields] OR "developing countr*" [All Fields] OR "resource-limited countries" [All Fields]OR Afghanistan [All Fields] OR "Guinea-Bissau" [All Fields] OR "Sierra Leone" [All Fields] OR "Burkina Faso" [All Fields] OR Haiti [All Fields] OR Somalia [All Fields] OR Burundi [All Fields] OR Korea [All Fields] OR "Democratic republic" [All Fields] OR "South Sudan" [All Fields] OR "Central African Republic" [All Fields] OR Liberia [All Fields] OR Sudan [All Fields] OR Chad [All Fields] OR Madagascar [All Fields] OR Syrian [All Fields] OR Congo [All Fields] OR Malawi [All Fields] OR Tajikistan [All Fields] OR Eritrea [All Fields] OR Mali [All Fields] OR Togo [All Fields] OR Ethiopia [All Fields] OR Mozambique [All Fields] OR Uganda [All Fields] OR Gambia [All Fields] OR Niger [All Fields] OR Yemen [All Fields] OR "Republic Guinea" [All Fields] OR Rwanda [All Fields] OR Angola [All Fields] OR Honduras [All Fields] OR "Papua New Guinea" [All Fields] OR Algeria [All Fields] OR India [All Fields] OR Philippines [All Fields] OR Bangladesh [All Fields] OR Kenya [All Fields] OR "São Tomé" [All Fields] OR Benin [All Fields] OR Kiribati [All Fields] OR Senegal [All Fields] OR Bhutan [All Fields] OR [All Fields] "Kyrgyz Republic"[All Fields] OR "Solomon Islands" [All Fields] OR Bolivia [All Fields] OR "Lao PDR"[All Fields] OR "Sri Lanka" [All Fields] OR "Cape Verde" [All Fields] OR Lesotho [All Fields] OR Tanzania [All Fields] OR Cambodia [All Fields] OR Mauritania [All Fields] OR "Timor-Leste" [All Fields] OR Cameroon [All Fields] OR Micronesia [All Fields] OR Tunisia [All Fields] OR Comoros [All Fields] OR Moldova [All Fields] OR Ukraine [All Fields] OR "Congo Republic" [All Fields] OR Mongolia [All Fields] OR Uzbekistan [All Fields] OR "Côte d'Ivoire" [All Fields] OR Morocco [All Fields] OR Vanuatu [All Fields] OR Djibouti [All Fields] OR Myanmar [All Fields] OR Vietnam [All Fields] OR Egypt [All Fields] OR Nepal [All Fields] OR "West Bank and Gaza" OR "El Salvador" [All Fields] OR Nicaragua [All Fields] OR Zambia [All Fields] OR Eswatini [All Fields] OR Nigeria [All Fields] OR Zimbabwe [All Fields] OR Ghana [All Fields] OR Pakistan [All Fields] OR Albania [All Fields] OR "American Samoa" [All Fields] OR Argentina [All Fields] OR Armenia [All Fields] OR Azerbaijan [All Fields] OR Belarus [All Fields] OR Belize [All Fields] OR "Bosnia and Herzegovina" [All Fields] OR Botswana [All Fields] OR Brazil [All Fields] OR Bulgaria [All Fields] OR China [All Fields] OR Colombia [All Fields] OR "Costa Rica" [All Fields] OR Cuba [All Fields] OR Dominica [All Fields] OR "Dominican Republic" [All Fields] OR "Equatorial Guinea" [All Fields] OR Ecuador [All Fields] OR Fiji [All Fields] OR Gabon [All Fields] OR Georgia [All Fields] OR Grenada [All Fields] OR Guatemala [All Fields] OR Guyana [All Fields] OR Indonesia [All Fields] OR Iran [All Fields] OR Iraq [All Fields] OR Jamaica [All Fields] OR Jordan [All Fields] OR Kazakhstan [All Fields] OR Kosovo [All Fields] OR Lebanon [All Fields] OR Libya [All Fields] OR Malaysia [All Fields] OR "Marshall Islands" [All Fields] OR Mexico [All Fields] OR Montenegro [All Fields] OR Namibia [All Fields] OR "North Macedonia" [All Fields] OR Paraguay [All Fields] OR Peru [All Fields] OR "Russian Federation" [All Fields] OR Samoa [All Fields] OR Serbia [All Fields] OR "South Africa" [All Fields] OR "St. Lucia" [All Fields] OR "St. Vincent and the Grenadines" [All Fields] OR Suriname [All Fields] OR Thailand [All Fields] OR Tonga [All Fields] OR Turkey [All Fields] OR Turkmenistan [All Fields] OR Tuvalu [All Fields] OR Venezuela [All Fields] OR ((Afghanistan [Mesh] OR "Guinea-Bissau" [Mesh] OR "Sierra Leone" [Mesh] OR "Burkina Faso" [Mesh] OR Haiti [Mesh] OR Somalia [Mesh] OR Burundi [Mesh] OR Korea [Mesh] OR "Democratic republic" OR "South Sudan" [Mesh] OR "Central African Republic" [Mesh] OR Liberia [Mesh] OR Sudan [Mesh] OR Chad [Mesh] OR Madagascar [Mesh] OR Syrian [Mesh] OR Congo [Mesh] OR Malawi [Mesh] OR Tajikistan [Mesh] OR Eritrea [Mesh] OR Mali [Mesh] OR Togo [Mesh] OR Ethiopia [Mesh] OR Mozambique [Mesh] OR Uganda [Mesh] OR Gambia [Mesh] OR Niger [Mesh] OR Yemen [Mesh] OR "Republic Guinea" OR Rwanda [Mesh] OR Angola [Mesh] OR Honduras [Mesh] OR "Papua New Guinea" [Mesh] OR Algeria [Mesh] OR India [Mesh] OR Philippines [Mesh] OR Bangladesh [Mesh] OR Kenya [Mesh] OR "São Tomé" OR Benin [Mesh] OR Kiribati [Mesh] OR Senegal [Mesh] OR Bhutan [Mesh] OR "Kyrgyz Republic" OR "Solomon Islands" OR Bolivia [Mesh] OR "Lao PDR" OR "Sri Lanka" [Mesh] OR "Cape Verde" OR Lesotho [Mesh] OR Tanzania [Mesh] OR Cambodia [Mesh] OR Mauritania [Mesh] OR "Timor-Leste" [Mesh] OR Cameroon [Mesh] OR Micronesia [Mesh] OR Tunisia [Mesh] OR Comoros [Mesh] OR Moldova [Mesh] OR Ukraine [Mesh] OR "Congo Republic" OR Mongolia [Mesh] OR Uzbekistan [Mesh] OR "Côte d'Ivoire" [Mesh] OR Morocco [Mesh] OR Vanuatu [Mesh] OR Djibouti [Mesh] OR Myanmar [Mesh] OR Vietnam [Mesh] OR Egypt [Mesh] OR Nepal [Mesh] OR "West Bank and Gaza" OR "El Salvador"[Mesh] OR Nicaragua [Mesh] OR Zambia [Mesh] OR Eswatini [Mesh] OR Nigeria [Mesh] OR Zimbabwe [Mesh] OR Ghana [Mesh] OR Pakistan [Mesh] OR Albania [Mesh] OR "American Samoa" [Mesh] OR Argentina [Mesh] OR Armenia [Mesh] OR Azerbaijan [Mesh] OR Belarus [Mesh] OR Belize [Mesh] OR "Bosnia and Herzegovina" [Mesh] OR Botswana [Mesh] OR Brazil [Mesh] OR Bulgaria [Mesh] OR China [Mesh] OR Colombia [Mesh] OR "Costa Rica" [Mesh] OR Cuba [Mesh] OR Dominica [Mesh] OR "Dominican Republic" [Mesh] OR "Equatorial Guinea" [Mesh] OR Ecuador [Mesh] OR Fiji [Mesh] OR Gabon [Mesh] OR Georgia [Mesh] OR Grenada [Mesh] OR Guatemala [Mesh] OR Guyana [Mesh] OR Indonesia [Mesh] OR Iran [Mesh] OR Iraq [Mesh] OR Jamaica [Mesh] OR Jordan [Mesh] OR Kazakhstan [Mesh] OR Kosovo [Mesh] OR Lebanon [Mesh] OR Libya [Mesh] OR Malaysia [Mesh] OR Mexico [Mesh] OR Montenegro [Mesh] OR Namibia [Mesh] OR Paraguay [Mesh] OR Peru [Mesh] OR Samoa [Mesh] OR Serbia [Mesh] OR "South Africa" [Mesh] OR Suriname [Mesh] OR Thailand [Mesh] OR Tonga [Mesh] OR Turkey [Mesh] OR Turkmenistan [Mesh] OR Tuvalu [Mesh] OR Venezuela [Mesh]))) |  |  | ("women"[All Fields] OR "women"[MeSH Terms] OR "reproductive age"[All Fields] OR "15-49 years"[All Fields]) AND ("disabili*"[All Fields] OR "disabili*"[MeSH Terms] OR "impairmen*"[All Fields] OR "physical disabilit*"[All Fields] OR "visual impairmen*"[All Fields] OR "visual loss"[All Fields] OR "blind"[All Fields] OR ("visually impaired persons"[MeSH Terms] OR "blindness"[MeSH Terms]) OR "hearing loss"[All Fields] OR "hearing loss"[MeSH Terms] OR "hearing impairmen*"[All Fields] OR "deaf"[All Fields] OR "intellectual disabili*"[All Fields] OR "intellectual disabili*"[MeSH Terms] OR "sensory disabili*"[All Fields]) AND ("Birth control"[All Fields] OR "family planning services"[All Fields] OR "family planning services"[MeSH Terms] OR "contraception behavior"[MeSH Terms] OR "contraception/psychology"[MeSH Terms] OR "contraception/utilization"[All Fields] OR "family plannin*"[All Fields] OR "contracepti*"[All Fields] OR "contracepti*"[MeSH Terms] OR "contraceptive agen*"[All Fields] OR "contraceptive agen*"[MeSH Terms] OR "contraceptive methods"[All Fields] OR "contraceptive device*"[MeSH Terms] OR "contraceptive devic*"[All Fields] OR "planned pregnanc*"[All Fields] OR "birth prevention*"[All Fields] OR "prevent pregnanc*"[All Fields] OR "birth interva*"[MeSH Terms] OR "birth interva*"[All Fields] OR "birth spacing"[All Fields] OR "pregnancy interval"[All Fields] OR "pregnancy spacing"[All Fields]) AND ("dynami*"[All Fields] OR "dynami*"[MeSH Terms] OR "utilizatio*"[All Fields] OR "utilizatio*"[MeSH Terms] OR "use"[All Fields] OR "practic*"[MeSH Terms] OR "practic*"[All Fields] OR "unmet nee*"[All Fields] OR "discontinuatio*"[All Fields] OR "dis continuatio*"[All Fields] OR "switchin*"[All Fields] OR "chang*"[All Fields] OR "chang*"[MeSH Terms]) AND (((("middle income countr*"[All Fields] OR "low income countr*"[All Fields] OR "developing countr*"[All Fields] OR "resource-limited countries"[All Fields] OR ("afghanistan"[MeSH Terms] OR "afghanistan"[All Fields] OR "afghanistan s"[All Fields]) OR "Guinea-Bissau"[All Fields] OR "Sierra Leone"[All Fields] OR "Burkina Faso"[All Fields] OR ("haiti"[MeSH Terms] OR "haiti"[All Fields] OR "haiti s"[All Fields]) OR ("somalia"[MeSH Terms] OR "somalia"[All Fields]) OR ("burundi"[MeSH Terms] OR "burundi"[All Fields]) OR ("korea"[MeSH Terms] OR "korea"[All Fields] OR "korea s"[All Fields] OR "koreas"[All Fields]) OR "Democratic republic"[All Fields] OR "South Sudan"[All Fields] OR "Central African Republic"[All Fields] OR ("liberia"[MeSH Terms] OR "liberia"[All Fields] OR "liberia s"[All Fields]) OR ("sudan"[MeSH Terms] OR "sudan"[All Fields] OR "sudans"[All Fields] OR "sudan s"[All Fields]) OR ("chad"[MeSH Terms] OR "chad"[All Fields]) OR ("madagascar"[MeSH Terms] OR "madagascar"[All Fields] OR "madagascar s"[All Fields]) OR ("mesocricetus"[MeSH Terms] OR "mesocricetus"[All Fields] OR "syrian"[All Fields] OR "syrians"[All Fields]) OR ("congo"[MeSH Terms] OR "congo"[All Fields]) OR ("malawi"[MeSH Terms] OR "malawi"[All Fields] OR "malawi s"[All Fields]) OR ("tajikistan"[MeSH Terms] OR "tajikistan"[All Fields]) OR ("eritrea"[MeSH Terms] OR "eritrea"[All Fields]) OR ("mali"[MeSH Terms] OR "mali"[All Fields]) OR ("togo"[MeSH Terms] OR "togo"[All Fields]) OR ("ethiopia"[MeSH Terms] OR "ethiopia"[All Fields] OR "ethiopia s"[All Fields]) OR ("mozambique"[MeSH Terms] OR "mozambique"[All Fields] OR "mozambique s"[All Fields]) OR ("uganda"[MeSH Terms] OR "uganda"[All Fields] OR "uganda s"[All Fields]) OR ("gambia"[MeSH Terms] OR "gambia"[All Fields] OR "gambia s"[All Fields]) OR ("niger"[MeSH Terms] OR "niger"[All Fields]) OR ("yemen"[MeSH Terms] OR "yemen"[All Fields]) OR "Republic Guinea"[All Fields] OR ("rwanda"[MeSH Terms] OR "rwanda"[All Fields] OR "rwanda s"[All Fields]) OR ("angola"[MeSH Terms] OR "angola"[All Fields] OR "angola s"[All Fields]) OR ("honduras"[MeSH Terms] OR "honduras"[All Fields]) OR "Papua New Guinea"[All Fields] OR ("algeria"[MeSH Terms] OR "algeria"[All Fields]) OR ("india"[MeSH Terms] OR "india"[All Fields] OR "india s"[All Fields] OR "indias"[All Fields]) OR ("philippine"[All Fields] OR "philippines"[MeSH Terms] OR "philippines"[All Fields]) OR ("bangladesh"[MeSH Terms] OR "bangladesh"[All Fields] OR "bangladesh s"[All Fields]) OR ("kenya"[MeSH Terms] OR "kenya"[All Fields] OR "kenya s"[All Fields]) OR "Sao Tome"[All Fields] OR ("benin"[MeSH Terms] OR "benin"[All Fields] OR "benin s"[All Fields]) OR ("micronesia"[MeSH Terms] OR "micronesia"[All Fields] OR "kiribati"[All Fields]) OR ("senegal"[MeSH Terms] OR "senegal"[All Fields] OR "senegal s"[All Fields]) OR ("bhutan"[MeSH Terms] OR "bhutan"[All Fields] OR "bhutan s"[All Fields])) AND "OR"[All Fields]) AND "Kyrgyz Republic"[All Fields]) OR "Solomon Islands"[All Fields] OR ("bolivia"[MeSH Terms] OR "bolivia"[All Fields]) OR "Lao PDR"[All Fields] OR "Sri Lanka"[All Fields] OR "Cape Verde"[All Fields] OR ("lesotho"[MeSH Terms] OR "lesotho"[All Fields]) OR ("tanzania"[MeSH Terms] OR "tanzania"[All Fields] OR "tanzania s"[All Fields]) OR ("cambodia"[MeSH Terms] OR "cambodia"[All Fields] OR "cambodia s"[All Fields]) OR ("mauritania"[MeSH Terms] OR "mauritania"[All Fields]) OR "Timor-Leste"[All Fields] OR ("cameroon"[MeSH Terms] OR "cameroon"[All Fields] OR "cameroons"[All Fields] OR "cameroon s"[All Fields]) OR ("micronesia"[MeSH Terms] OR "micronesia"[All Fields]) OR ("tunisia"[MeSH Terms] OR "tunisia"[All Fields]) OR ("comoros"[MeSH Terms] OR "comoros"[All Fields] OR "comoro"[All Fields]) OR ("moldova"[MeSH Terms] OR "moldova"[All Fields]) OR ("ukraine"[MeSH Terms] OR "ukraine"[All Fields] OR "ukraine s"[All Fields]) OR "Congo Republic"[All Fields] OR ("mongolia"[MeSH Terms] OR "mongolia"[All Fields] OR "mongolia s"[All Fields]) OR ("uzbekistan"[MeSH Terms] OR "uzbekistan"[All Fields]) OR "Cote d'Ivoire"[All Fields] OR ("morocco"[MeSH Terms] OR "morocco"[All Fields]) OR ("vanuatu"[MeSH Terms] OR "vanuatu"[All Fields]) OR ("djibouti"[MeSH Terms] OR "djibouti"[All Fields]) OR ("myanmar"[MeSH Terms] OR "myanmar"[All Fields] OR "myanmar s"[All Fields] OR "myanmars"[All Fields]) OR ("vietnam"[MeSH Terms] OR "vietnam"[All Fields] OR "vietnam s"[All Fields]) OR ("egypt"[MeSH Terms] OR "egypt"[All Fields] OR "egypt s"[All Fields]) OR ("nepal"[MeSH Terms] OR "nepal"[All Fields] OR "nepal s"[All Fields]) OR "West Bank and Gaza"[All Fields] OR "El Salvador"[All Fields] OR ("nicaragua"[MeSH Terms] OR "nicaragua"[All Fields] OR "nicaragua s"[All Fields]) OR ("zambia"[MeSH Terms] OR "zambia"[All Fields] OR "zambia s"[All Fields]) OR ("eswatini"[MeSH Terms] OR "eswatini"[All Fields]) OR ("nigeria"[MeSH Terms] OR "nigeria"[All Fields] OR "nigeria s"[All Fields]) OR ("zimbabwe"[MeSH Terms] OR "zimbabwe"[All Fields] OR "zimbabwe s"[All Fields]) OR ("ghana"[MeSH Terms] OR "ghana"[All Fields] OR "ghana s"[All Fields]) OR ("pakistan"[MeSH Terms] OR "pakistan"[All Fields] OR "pakistan s"[All Fields]) OR ("albania"[MeSH Terms] OR "albania"[All Fields]) OR "American Samoa"[All Fields] OR ("argentina"[MeSH Terms] OR "argentina"[All Fields] OR "argentina s"[All Fields] OR "argentinae"[All Fields]) OR ("armenia"[MeSH Terms] OR "armenia"[All Fields]) OR ("azerbaijan"[MeSH Terms] OR "azerbaijan"[All Fields]) OR ("republic of belarus"[MeSH Terms] OR ("republic"[All Fields] AND "belarus"[All Fields]) OR "republic of belarus"[All Fields] OR "belarus"[All Fields]) OR ("belize"[MeSH Terms] OR "belize"[All Fields]) OR "Bosnia and Herzegovina"[All Fields] OR ("botswana"[MeSH Terms] OR "botswana"[All Fields] OR "botswana s"[All Fields]) OR ("brazil"[MeSH Terms] OR "brazil"[All Fields] OR "brazil s"[All Fields] OR "brazils"[All Fields]) OR ("bulgaria"[MeSH Terms] OR "bulgaria"[All Fields]) OR ("china"[MeSH Terms] OR "china"[All Fields] OR "china s"[All Fields] OR "chinas"[All Fields]) OR ("colombia"[MeSH Terms] OR "colombia"[All Fields] OR "colombia s"[All Fields]) OR "Costa Rica"[All Fields] OR ("cuba"[MeSH Terms] OR "cuba"[All Fields]) OR ("dominica"[MeSH Terms] OR "dominica"[All Fields]) OR "Dominican Republic"[All Fields] OR "Equatorial Guinea"[All Fields] OR ("ecuador"[MeSH Terms] OR "ecuador"[All Fields] OR "ecuador s"[All Fields]) OR ("fiji"[MeSH Terms] OR "fiji"[All Fields]) OR ("gabon"[MeSH Terms] OR "gabon"[All Fields]) OR ("georgia"[MeSH Terms] OR "georgia"[All Fields] OR "georgia republic"[MeSH Terms] OR ("georgia"[All Fields] AND "republic"[All Fields]) OR "georgia republic"[All Fields] OR "georgia s"[All Fields]) OR ("grenada"[MeSH Terms] OR "grenada"[All Fields]) OR ("guatemala"[MeSH Terms] OR "guatemala"[All Fields] OR "guatemala s"[All Fields]) OR ("guyana"[MeSH Terms] OR "guyana"[All Fields]) OR ("indonesia"[MeSH Terms] OR "indonesia"[All Fields] OR "indonesia s"[All Fields] OR "indonesias"[All Fields]) OR ("iran"[MeSH Terms] OR "iran"[All Fields]) OR ("iraq"[MeSH Terms] OR "iraq"[All Fields]) OR ("jamaica"[MeSH Terms] OR "jamaica"[All Fields] OR "jamaica s"[All Fields]) OR ("jordan"[MeSH Terms] OR "jordan"[All Fields]) OR ("kazakhstan"[MeSH Terms] OR "kazakhstan"[All Fields] OR "kazakhstan s"[All Fields]) OR ("kosovo"[MeSH Terms] OR "kosovo"[All Fields] OR "kosovo s"[All Fields]) OR ("lebanon"[MeSH Terms] OR "lebanon"[All Fields] OR "lebanon s"[All Fields]) OR ("libya"[MeSH Terms] OR "libya"[All Fields]) OR ("malaysia"[MeSH Terms] OR "malaysia"[All Fields] OR "malaysia s"[All Fields]) OR "Marshall Islands"[All Fields] OR ("mexico"[MeSH Terms] OR "mexico"[All Fields] OR "mexico s"[All Fields] OR "mexicos"[All Fields]) OR ("montenegro"[MeSH Terms] OR "montenegro"[All Fields]) OR ("namibia"[MeSH Terms] OR "namibia"[All Fields] OR "namibia s"[All Fields]) OR "North Macedonia"[All Fields] OR ("paraguai"[All Fields] OR "paraguay"[MeSH Terms] OR "paraguay"[All Fields]) OR ("peru"[MeSH Terms] OR "peru"[All Fields]) OR "Russian Federation"[All Fields] OR ("samoa"[MeSH Terms] OR "samoa"[All Fields] OR "samoas"[All Fields]) OR ("serbia"[MeSH Terms] OR "serbia"[All Fields]) OR "South Africa"[All Fields] OR "st lucia"[All Fields] OR "st vincent and the grenadines"[All Fields] OR ("suriname"[MeSH Terms] OR "suriname"[All Fields] OR "surinam"[All Fields]) OR ("thailand"[MeSH Terms] OR "thailand"[All Fields] OR "thailand s"[All Fields]) OR ("tonga"[MeSH Terms] OR "tonga"[All Fields] OR "tonga s"[All Fields]) OR ("turkey"[MeSH Terms] OR "turkey"[All Fields] OR "turkey s"[All Fields] OR "turkeys"[MeSH Terms] OR "turkeys"[All Fields]) OR ("turkmenistan"[MeSH Terms] OR "turkmenistan"[All Fields]) OR ("micronesia"[MeSH Terms] OR "micronesia"[All Fields] OR "tuvalu"[All Fields]) OR ("venezuela"[MeSH Terms] OR "venezuela"[All Fields] OR "venezuela s"[All Fields]) OR ("afghanistan"[MeSH Terms] OR "Guinea-Bissau"[MeSH Terms] OR "Sierra Leone"[MeSH Terms] OR "Burkina Faso"[MeSH Terms] OR "haiti"[MeSH Terms] OR "somalia"[MeSH Terms] OR "burundi"[MeSH Terms] OR "korea"[MeSH Terms] OR "Democratic republic"[All Fields] OR "South Sudan"[MeSH Terms] OR "Central African Republic"[MeSH Terms] OR "liberia"[MeSH Terms] OR "sudan"[MeSH Terms] OR "chad"[MeSH Terms] OR "madagascar"[MeSH Terms] OR "mesocricetus"[MeSH Terms] OR "congo"[MeSH Terms] OR "malawi"[MeSH Terms] OR "tajikistan"[MeSH Terms] OR "eritrea"[MeSH Terms] OR "mali"[MeSH Terms] OR "togo"[MeSH Terms] OR "ethiopia"[MeSH Terms] OR "mozambique"[MeSH Terms] OR "uganda"[MeSH Terms] OR "gambia"[MeSH Terms] OR "niger"[MeSH Terms] OR "yemen"[MeSH Terms] OR "Republic Guinea"[All Fields] OR "rwanda"[MeSH Terms] OR "angola"[MeSH Terms] OR "honduras"[MeSH Terms] OR "Papua New Guinea"[MeSH Terms] OR "algeria"[MeSH Terms] OR "india"[MeSH Terms] OR "philippines"[MeSH Terms] OR "bangladesh"[MeSH Terms] OR "kenya"[MeSH Terms] OR "Sao Tome"[All Fields] OR "benin"[MeSH Terms] OR "micronesia"[MeSH Terms] OR "senegal"[MeSH Terms] OR "bhutan"[MeSH Terms] OR "Kyrgyz Republic"[All Fields] OR "Solomon Islands"[All Fields] OR "bolivia"[MeSH Terms] OR "Lao PDR"[All Fields] OR "Sri Lanka"[MeSH Terms] OR "Cape Verde"[All Fields] OR "lesotho"[MeSH Terms] OR "tanzania"[MeSH Terms] OR "cambodia"[MeSH Terms] OR "mauritania"[MeSH Terms] OR "Timor-Leste"[MeSH Terms] OR "cameroon"[MeSH Terms] OR "micronesia"[MeSH Terms] OR "tunisia"[MeSH Terms] OR "comoros"[MeSH Terms] OR "moldova"[MeSH Terms] OR "ukraine"[MeSH Terms] OR "Congo Republic"[All Fields] OR "mongolia"[MeSH Terms] OR "uzbekistan"[MeSH Terms] OR "Cote d'Ivoire"[MeSH Terms] OR "morocco"[MeSH Terms] OR "vanuatu"[MeSH Terms] OR "djibouti"[MeSH Terms] OR "myanmar"[MeSH Terms] OR "vietnam"[MeSH Terms] OR "egypt"[MeSH Terms] OR "nepal"[MeSH Terms] OR "West Bank and Gaza"[All Fields] OR "El Salvador"[MeSH Terms] OR "nicaragua"[MeSH Terms] OR "zambia"[MeSH Terms] OR "eswatini"[MeSH Terms] OR "nigeria"[MeSH Terms] OR "zimbabwe"[MeSH Terms] OR "ghana"[MeSH Terms] OR "pakistan"[MeSH Terms] OR "albania"[MeSH Terms] OR "American Samoa"[MeSH Terms] OR "argentina"[MeSH Terms] OR "armenia"[MeSH Terms] OR "azerbaijan"[MeSH Terms] OR "republic of belarus"[MeSH Terms] OR "belize"[MeSH Terms] OR "Bosnia and Herzegovina"[MeSH Terms] OR "botswana"[MeSH Terms] OR "brazil"[MeSH Terms] OR "bulgaria"[MeSH Terms] OR "china"[MeSH Terms] OR "colombia"[MeSH Terms] OR "Costa Rica"[MeSH Terms] OR "cuba"[MeSH Terms] OR "dominica"[MeSH Terms] OR "Dominican Republic"[MeSH Terms] OR "Equatorial Guinea"[MeSH Terms] OR "ecuador"[MeSH Terms] OR "fiji"[MeSH Terms] OR "gabon"[MeSH Terms] OR ("georgia"[MeSH Terms] OR "georgia republic"[MeSH Terms]) OR "grenada"[MeSH Terms] OR "guatemala"[MeSH Terms] OR "guyana"[MeSH Terms] OR "indonesia"[MeSH Terms] OR "iran"[MeSH Terms] OR "iraq"[MeSH Terms] OR "jamaica"[MeSH Terms] OR "jordan"[MeSH Terms] OR "kazakhstan"[MeSH Terms] OR "kosovo"[MeSH Terms] OR "lebanon"[MeSH Terms] OR "libya"[MeSH Terms] OR "malaysia"[MeSH Terms] OR "mexico"[MeSH Terms] OR "montenegro"[MeSH Terms] OR "namibia"[MeSH Terms] OR "paraguay"[MeSH Terms] OR "peru"[MeSH Terms] OR "samoa"[MeSH Terms] OR "serbia"[MeSH Terms] OR "South Africa"[MeSH Terms] OR "suriname"[MeSH Terms] OR "thailand"[MeSH Terms] OR "tonga"[MeSH Terms] OR "turkey"[MeSH Terms] OR "turkmenistan"[MeSH Terms] OR "micronesia"[MeSH Terms] OR "venezuela"[MeSH Terms])) | 287 |
| 12 | "middle income countr*" [All Fields] OR "low income countr*" [All Fields] OR "developing countr*" [All Fields] OR "resource-limited countries" [All Fields]OR Afghanistan [All Fields] OR "Guinea-Bissau" [All Fields] OR "Sierra Leone" [All Fields] OR "Burkina Faso" [All Fields] OR Haiti [All Fields] OR Somalia [All Fields] OR Burundi [All Fields] OR Korea [All Fields] OR "Democratic republic" [All Fields] OR "South Sudan" [All Fields] OR "Central African Republic" [All Fields] OR Liberia [All Fields] OR Sudan [All Fields] OR Chad [All Fields] OR Madagascar [All Fields] OR Syrian [All Fields] OR Congo [All Fields] OR Malawi [All Fields] OR Tajikistan [All Fields] OR Eritrea [All Fields] OR Mali [All Fields] OR Togo [All Fields] OR Ethiopia [All Fields] OR Mozambique [All Fields] OR Uganda [All Fields] OR Gambia [All Fields] OR Niger [All Fields] OR Yemen [All Fields] OR "Republic Guinea" [All Fields] OR Rwanda [All Fields] OR Angola [All Fields] OR Honduras [All Fields] OR "Papua New Guinea" [All Fields] OR Algeria [All Fields] OR India [All Fields] OR Philippines [All Fields] OR Bangladesh [All Fields] OR Kenya [All Fields] OR "São Tomé" [All Fields] OR Benin [All Fields] OR Kiribati [All Fields] OR Senegal [All Fields] OR Bhutan [All Fields] OR [All Fields] "Kyrgyz Republic"[All Fields] OR "Solomon Islands" [All Fields] OR Bolivia [All Fields] OR "Lao PDR"[All Fields] OR "Sri Lanka" [All Fields] OR "Cape Verde" [All Fields] OR Lesotho [All Fields] OR Tanzania [All Fields] OR Cambodia [All Fields] OR Mauritania [All Fields] OR "Timor-Leste" [All Fields] OR Cameroon [All Fields] OR Micronesia [All Fields] OR Tunisia [All Fields] OR Comoros [All Fields] OR Moldova [All Fields] OR Ukraine [All Fields] OR "Congo Republic" [All Fields] OR Mongolia [All Fields] OR Uzbekistan [All Fields] OR "Côte d'Ivoire" [All Fields] OR Morocco [All Fields] OR Vanuatu [All Fields] OR Djibouti [All Fields] OR Myanmar [All Fields] OR Vietnam [All Fields] OR Egypt [All Fields] OR Nepal [All Fields] OR "West Bank and Gaza" OR "El Salvador" [All Fields] OR Nicaragua [All Fields] OR Zambia [All Fields] OR Eswatini [All Fields] OR Nigeria [All Fields] OR Zimbabwe [All Fields] OR Ghana [All Fields] OR Pakistan [All Fields] OR Albania [All Fields] OR "American Samoa" [All Fields] OR Argentina [All Fields] OR Armenia [All Fields] OR Azerbaijan [All Fields] OR Belarus [All Fields] OR Belize [All Fields] OR "Bosnia and Herzegovina" [All Fields] OR Botswana [All Fields] OR Brazil [All Fields] OR Bulgaria [All Fields] OR China [All Fields] OR Colombia [All Fields] OR "Costa Rica" [All Fields] OR Cuba [All Fields] OR Dominica [All Fields] OR "Dominican Republic" [All Fields] OR "Equatorial Guinea" [All Fields] OR Ecuador [All Fields] OR Fiji [All Fields] OR Gabon [All Fields] OR Georgia [All Fields] OR Grenada [All Fields] OR Guatemala [All Fields] OR Guyana [All Fields] OR Indonesia [All Fields] OR Iran [All Fields] OR Iraq [All Fields] OR Jamaica [All Fields] OR Jordan [All Fields] OR Kazakhstan [All Fields] OR Kosovo [All Fields] OR Lebanon [All Fields] OR Libya [All Fields] OR Malaysia [All Fields] OR "Marshall Islands" [All Fields] OR Mexico [All Fields] OR Montenegro [All Fields] OR Namibia [All Fields] OR "North Macedonia" [All Fields] OR Paraguay [All Fields] OR Peru [All Fields] OR "Russian Federation" [All Fields] OR Samoa [All Fields] OR Serbia [All Fields] OR "South Africa" [All Fields] OR "St. Lucia" [All Fields] OR "St. Vincent and the Grenadines" [All Fields] OR Suriname [All Fields] OR Thailand [All Fields] OR Tonga [All Fields] OR Turkey [All Fields] OR Turkmenistan [All Fields] OR Tuvalu [All Fields] OR Venezuela [All Fields] OR ((Afghanistan [Mesh] OR "Guinea-Bissau" [Mesh] OR "Sierra Leone" [Mesh] OR "Burkina Faso" [Mesh] OR Haiti [Mesh] OR Somalia [Mesh] OR Burundi [Mesh] OR Korea [Mesh] OR "Democratic republic" OR "South Sudan" [Mesh] OR "Central African Republic" [Mesh] OR Liberia [Mesh] OR Sudan [Mesh] OR Chad [Mesh] OR Madagascar [Mesh] OR Syrian [Mesh] OR Congo [Mesh] OR Malawi [Mesh] OR Tajikistan [Mesh] OR Eritrea [Mesh] OR Mali [Mesh] OR Togo [Mesh] OR Ethiopia [Mesh] OR Mozambique [Mesh] OR Uganda [Mesh] OR Gambia [Mesh] OR Niger [Mesh] OR Yemen [Mesh] OR "Republic Guinea" OR Rwanda [Mesh] OR Angola [Mesh] OR Honduras [Mesh] OR "Papua New Guinea" [Mesh] OR Algeria [Mesh] OR India [Mesh] OR Philippines [Mesh] OR Bangladesh [Mesh] OR Kenya [Mesh] OR "São Tomé" OR Benin [Mesh] OR Kiribati [Mesh] OR Senegal [Mesh] OR Bhutan [Mesh] OR "Kyrgyz Republic" OR "Solomon Islands" OR Bolivia [Mesh] OR "Lao PDR" OR "Sri Lanka" [Mesh] OR "Cape Verde" OR Lesotho [Mesh] OR Tanzania [Mesh] OR Cambodia [Mesh] OR Mauritania [Mesh] OR "Timor-Leste" [Mesh] OR Cameroon [Mesh] OR Micronesia [Mesh] OR Tunisia [Mesh] OR Comoros [Mesh] OR Moldova [Mesh] OR Ukraine [Mesh] OR "Congo Republic" OR Mongolia [Mesh] OR Uzbekistan [Mesh] OR "Côte d'Ivoire" [Mesh] OR Morocco [Mesh] OR Vanuatu [Mesh] OR Djibouti [Mesh] OR Myanmar [Mesh] OR Vietnam [Mesh] OR Egypt [Mesh] OR Nepal [Mesh] OR "West Bank and Gaza" OR "El Salvador"[Mesh] OR Nicaragua [Mesh] OR Zambia [Mesh] OR Eswatini [Mesh] OR Nigeria [Mesh] OR Zimbabwe [Mesh] OR Ghana [Mesh] OR Pakistan [Mesh] OR Albania [Mesh] OR "American Samoa" [Mesh] OR Argentina [Mesh] OR Armenia [Mesh] OR Azerbaijan [Mesh] OR Belarus [Mesh] OR Belize [Mesh] OR "Bosnia and Herzegovina" [Mesh] OR Botswana [Mesh] OR Brazil [Mesh] OR Bulgaria [Mesh] OR China [Mesh] OR Colombia [Mesh] OR "Costa Rica" [Mesh] OR Cuba [Mesh] OR Dominica [Mesh] OR "Dominican Republic" [Mesh] OR "Equatorial Guinea" [Mesh] OR Ecuador [Mesh] OR Fiji [Mesh] OR Gabon [Mesh] OR Georgia [Mesh] OR Grenada [Mesh] OR Guatemala [Mesh] OR Guyana [Mesh] OR Indonesia [Mesh] OR Iran [Mesh] OR Iraq [Mesh] OR Jamaica [Mesh] OR Jordan [Mesh] OR Kazakhstan [Mesh] OR Kosovo [Mesh] OR Lebanon [Mesh] OR Libya [Mesh] OR Malaysia [Mesh] OR Mexico [Mesh] OR Montenegro [Mesh] OR Namibia [Mesh] OR Paraguay [Mesh] OR Peru [Mesh] OR Samoa [Mesh] OR Serbia [Mesh] OR "South Africa" [Mesh] OR Suriname [Mesh] OR Thailand [Mesh] OR Tonga [Mesh] OR Turkey [Mesh] OR Turkmenistan [Mesh] OR Tuvalu [Mesh] OR Venezuela [Mesh])) |  |  | ((("middle income countr*"[All Fields] OR "low income countr*"[All Fields] OR "developing countr*"[All Fields] OR "resource-limited countries"[All Fields] OR ("afghanistan"[MeSH Terms] OR "afghanistan"[All Fields] OR "afghanistan s"[All Fields]) OR "Guinea-Bissau"[All Fields] OR "Sierra Leone"[All Fields] OR "Burkina Faso"[All Fields] OR ("haiti"[MeSH Terms] OR "haiti"[All Fields] OR "haiti s"[All Fields]) OR ("somalia"[MeSH Terms] OR "somalia"[All Fields]) OR ("burundi"[MeSH Terms] OR "burundi"[All Fields]) OR ("korea"[MeSH Terms] OR "korea"[All Fields] OR "korea s"[All Fields] OR "koreas"[All Fields]) OR "Democratic republic"[All Fields] OR "South Sudan"[All Fields] OR "Central African Republic"[All Fields] OR ("liberia"[MeSH Terms] OR "liberia"[All Fields] OR "liberia s"[All Fields]) OR ("sudan"[MeSH Terms] OR "sudan"[All Fields] OR "sudans"[All Fields] OR "sudan s"[All Fields]) OR ("chad"[MeSH Terms] OR "chad"[All Fields]) OR ("madagascar"[MeSH Terms] OR "madagascar"[All Fields] OR "madagascar s"[All Fields]) OR ("mesocricetus"[MeSH Terms] OR "mesocricetus"[All Fields] OR "syrian"[All Fields] OR "syrians"[All Fields]) OR ("congo"[MeSH Terms] OR "congo"[All Fields]) OR ("malawi"[MeSH Terms] OR "malawi"[All Fields] OR "malawi s"[All Fields]) OR ("tajikistan"[MeSH Terms] OR "tajikistan"[All Fields]) OR ("eritrea"[MeSH Terms] OR "eritrea"[All Fields]) OR ("mali"[MeSH Terms] OR "mali"[All Fields]) OR ("togo"[MeSH Terms] OR "togo"[All Fields]) OR ("ethiopia"[MeSH Terms] OR "ethiopia"[All Fields] OR "ethiopia s"[All Fields]) OR ("mozambique"[MeSH Terms] OR "mozambique"[All Fields] OR "mozambique s"[All Fields]) OR ("uganda"[MeSH Terms] OR "uganda"[All Fields] OR "uganda s"[All Fields]) OR ("gambia"[MeSH Terms] OR "gambia"[All Fields] OR "gambia s"[All Fields]) OR ("niger"[MeSH Terms] OR "niger"[All Fields]) OR ("yemen"[MeSH Terms] OR "yemen"[All Fields]) OR "Republic Guinea"[All Fields] OR ("rwanda"[MeSH Terms] OR "rwanda"[All Fields] OR "rwanda s"[All Fields]) OR ("angola"[MeSH Terms] OR "angola"[All Fields] OR "angola s"[All Fields]) OR ("honduras"[MeSH Terms] OR "honduras"[All Fields]) OR "Papua New Guinea"[All Fields] OR ("algeria"[MeSH Terms] OR "algeria"[All Fields]) OR ("india"[MeSH Terms] OR "india"[All Fields] OR "india s"[All Fields] OR "indias"[All Fields]) OR ("philippine"[All Fields] OR "philippines"[MeSH Terms] OR "philippines"[All Fields]) OR ("bangladesh"[MeSH Terms] OR "bangladesh"[All Fields] OR "bangladesh s"[All Fields]) OR ("kenya"[MeSH Terms] OR "kenya"[All Fields] OR "kenya s"[All Fields]) OR "Sao Tome"[All Fields] OR ("benin"[MeSH Terms] OR "benin"[All Fields] OR "benin s"[All Fields]) OR ("micronesia"[MeSH Terms] OR "micronesia"[All Fields] OR "kiribati"[All Fields]) OR ("senegal"[MeSH Terms] OR "senegal"[All Fields] OR "senegal s"[All Fields]) OR ("bhutan"[MeSH Terms] OR "bhutan"[All Fields] OR "bhutan s"[All Fields])) AND "OR"[All Fields]) AND "Kyrgyz Republic"[All Fields]) OR "Solomon Islands"[All Fields] OR ("bolivia"[MeSH Terms] OR "bolivia"[All Fields]) OR "Lao PDR"[All Fields] OR "Sri Lanka"[All Fields] OR "Cape Verde"[All Fields] OR ("lesotho"[MeSH Terms] OR "lesotho"[All Fields]) OR ("tanzania"[MeSH Terms] OR "tanzania"[All Fields] OR "tanzania s"[All Fields]) OR ("cambodia"[MeSH Terms] OR "cambodia"[All Fields] OR "cambodia s"[All Fields]) OR ("mauritania"[MeSH Terms] OR "mauritania"[All Fields]) OR "Timor-Leste"[All Fields] OR ("cameroon"[MeSH Terms] OR "cameroon"[All Fields] OR "cameroons"[All Fields] OR "cameroon s"[All Fields]) OR ("micronesia"[MeSH Terms] OR "micronesia"[All Fields]) OR ("tunisia"[MeSH Terms] OR "tunisia"[All Fields]) OR ("comoros"[MeSH Terms] OR "comoros"[All Fields] OR "comoro"[All Fields]) OR ("moldova"[MeSH Terms] OR "moldova"[All Fields]) OR ("ukraine"[MeSH Terms] OR "ukraine"[All Fields] OR "ukraine s"[All Fields]) OR "Congo Republic"[All Fields] OR ("mongolia"[MeSH Terms] OR "mongolia"[All Fields] OR "mongolia s"[All Fields]) OR ("uzbekistan"[MeSH Terms] OR "uzbekistan"[All Fields]) OR "Cote d'Ivoire"[All Fields] OR ("morocco"[MeSH Terms] OR "morocco"[All Fields]) OR ("vanuatu"[MeSH Terms] OR "vanuatu"[All Fields]) OR ("djibouti"[MeSH Terms] OR "djibouti"[All Fields]) OR ("myanmar"[MeSH Terms] OR "myanmar"[All Fields] OR "myanmar s"[All Fields] OR "myanmars"[All Fields]) OR ("vietnam"[MeSH Terms] OR "vietnam"[All Fields] OR "vietnam s"[All Fields]) OR ("egypt"[MeSH Terms] OR "egypt"[All Fields] OR "egypt s"[All Fields]) OR ("nepal"[MeSH Terms] OR "nepal"[All Fields] OR "nepal s"[All Fields]) OR "West Bank and Gaza"[All Fields] OR "El Salvador"[All Fields] OR ("nicaragua"[MeSH Terms] OR "nicaragua"[All Fields] OR "nicaragua s"[All Fields]) OR ("zambia"[MeSH Terms] OR "zambia"[All Fields] OR "zambia s"[All Fields]) OR ("eswatini"[MeSH Terms] OR "eswatini"[All Fields]) OR ("nigeria"[MeSH Terms] OR "nigeria"[All Fields] OR "nigeria s"[All Fields]) OR ("zimbabwe"[MeSH Terms] OR "zimbabwe"[All Fields] OR "zimbabwe s"[All Fields]) OR ("ghana"[MeSH Terms] OR "ghana"[All Fields] OR "ghana s"[All Fields]) OR ("pakistan"[MeSH Terms] OR "pakistan"[All Fields] OR "pakistan s"[All Fields]) OR ("albania"[MeSH Terms] OR "albania"[All Fields]) OR "American Samoa"[All Fields] OR ("argentina"[MeSH Terms] OR "argentina"[All Fields] OR "argentina s"[All Fields] OR "argentinae"[All Fields]) OR ("armenia"[MeSH Terms] OR "armenia"[All Fields]) OR ("azerbaijan"[MeSH Terms] OR "azerbaijan"[All Fields]) OR ("republic of belarus"[MeSH Terms] OR ("republic"[All Fields] AND "belarus"[All Fields]) OR "republic of belarus"[All Fields] OR "belarus"[All Fields]) OR ("belize"[MeSH Terms] OR "belize"[All Fields]) OR "Bosnia and Herzegovina"[All Fields] OR ("botswana"[MeSH Terms] OR "botswana"[All Fields] OR "botswana s"[All Fields]) OR ("brazil"[MeSH Terms] OR "brazil"[All Fields] OR "brazil s"[All Fields] OR "brazils"[All Fields]) OR ("bulgaria"[MeSH Terms] OR "bulgaria"[All Fields]) OR ("china"[MeSH Terms] OR "china"[All Fields] OR "china s"[All Fields] OR "chinas"[All Fields]) OR ("colombia"[MeSH Terms] OR "colombia"[All Fields] OR "colombia s"[All Fields]) OR "Costa Rica"[All Fields] OR ("cuba"[MeSH Terms] OR "cuba"[All Fields]) OR ("dominica"[MeSH Terms] OR "dominica"[All Fields]) OR "Dominican Republic"[All Fields] OR "Equatorial Guinea"[All Fields] OR ("ecuador"[MeSH Terms] OR "ecuador"[All Fields] OR "ecuador s"[All Fields]) OR ("fiji"[MeSH Terms] OR "fiji"[All Fields]) OR ("gabon"[MeSH Terms] OR "gabon"[All Fields]) OR ("georgia"[MeSH Terms] OR "georgia"[All Fields] OR "georgia republic"[MeSH Terms] OR ("georgia"[All Fields] AND "republic"[All Fields]) OR "georgia republic"[All Fields] OR "georgia s"[All Fields]) OR ("grenada"[MeSH Terms] OR "grenada"[All Fields]) OR ("guatemala"[MeSH Terms] OR "guatemala"[All Fields] OR "guatemala s"[All Fields]) OR ("guyana"[MeSH Terms] OR "guyana"[All Fields]) OR ("indonesia"[MeSH Terms] OR "indonesia"[All Fields] OR "indonesia s"[All Fields] OR "indonesias"[All Fields]) OR ("iran"[MeSH Terms] OR "iran"[All Fields]) OR ("iraq"[MeSH Terms] OR "iraq"[All Fields]) OR ("jamaica"[MeSH Terms] OR "jamaica"[All Fields] OR "jamaica s"[All Fields]) OR ("jordan"[MeSH Terms] OR "jordan"[All Fields]) OR ("kazakhstan"[MeSH Terms] OR "kazakhstan"[All Fields] OR "kazakhstan s"[All Fields]) OR ("kosovo"[MeSH Terms] OR "kosovo"[All Fields] OR "kosovo s"[All Fields]) OR ("lebanon"[MeSH Terms] OR "lebanon"[All Fields] OR "lebanon s"[All Fields]) OR ("libya"[MeSH Terms] OR "libya"[All Fields]) OR ("malaysia"[MeSH Terms] OR "malaysia"[All Fields] OR "malaysia s"[All Fields]) OR "Marshall Islands"[All Fields] OR ("mexico"[MeSH Terms] OR "mexico"[All Fields] OR "mexico s"[All Fields] OR "mexicos"[All Fields]) OR ("montenegro"[MeSH Terms] OR "montenegro"[All Fields]) OR ("namibia"[MeSH Terms] OR "namibia"[All Fields] OR "namibia s"[All Fields]) OR "North Macedonia"[All Fields] OR ("paraguai"[All Fields] OR "paraguay"[MeSH Terms] OR "paraguay"[All Fields]) OR ("peru"[MeSH Terms] OR "peru"[All Fields]) OR "Russian Federation"[All Fields] OR ("samoa"[MeSH Terms] OR "samoa"[All Fields] OR "samoas"[All Fields]) OR ("serbia"[MeSH Terms] OR "serbia"[All Fields]) OR "South Africa"[All Fields] OR "st lucia"[All Fields] OR "st vincent and the grenadines"[All Fields] OR ("suriname"[MeSH Terms] OR "suriname"[All Fields] OR "surinam"[All Fields]) OR ("thailand"[MeSH Terms] OR "thailand"[All Fields] OR "thailand s"[All Fields]) OR ("tonga"[MeSH Terms] OR "tonga"[All Fields] OR "tonga s"[All Fields]) OR ("turkey"[MeSH Terms] OR "turkey"[All Fields] OR "turkey s"[All Fields] OR "turkeys"[MeSH Terms] OR "turkeys"[All Fields]) OR ("turkmenistan"[MeSH Terms] OR "turkmenistan"[All Fields]) OR ("micronesia"[MeSH Terms] OR "micronesia"[All Fields] OR "tuvalu"[All Fields]) OR ("venezuela"[MeSH Terms] OR "venezuela"[All Fields] OR "venezuela s"[All Fields]) OR ("afghanistan"[MeSH Terms] OR "Guinea-Bissau"[MeSH Terms] OR "Sierra Leone"[MeSH Terms] OR "Burkina Faso"[MeSH Terms] OR "haiti"[MeSH Terms] OR "somalia"[MeSH Terms] OR "burundi"[MeSH Terms] OR "korea"[MeSH Terms] OR "Democratic republic"[All Fields] OR "South Sudan"[MeSH Terms] OR "Central African Republic"[MeSH Terms] OR "liberia"[MeSH Terms] OR "sudan"[MeSH Terms] OR "chad"[MeSH Terms] OR "madagascar"[MeSH Terms] OR "mesocricetus"[MeSH Terms] OR "congo"[MeSH Terms] OR "malawi"[MeSH Terms] OR "tajikistan"[MeSH Terms] OR "eritrea"[MeSH Terms] OR "mali"[MeSH Terms] OR "togo"[MeSH Terms] OR "ethiopia"[MeSH Terms] OR "mozambique"[MeSH Terms] OR "uganda"[MeSH Terms] OR "gambia"[MeSH Terms] OR "niger"[MeSH Terms] OR "yemen"[MeSH Terms] OR "Republic Guinea"[All Fields] OR "rwanda"[MeSH Terms] OR "angola"[MeSH Terms] OR "honduras"[MeSH Terms] OR "Papua New Guinea"[MeSH Terms] OR "algeria"[MeSH Terms] OR "india"[MeSH Terms] OR "philippines"[MeSH Terms] OR "bangladesh"[MeSH Terms] OR "kenya"[MeSH Terms] OR "Sao Tome"[All Fields] OR "benin"[MeSH Terms] OR "micronesia"[MeSH Terms] OR "senegal"[MeSH Terms] OR "bhutan"[MeSH Terms] OR "Kyrgyz Republic"[All Fields] OR "Solomon Islands"[All Fields] OR "bolivia"[MeSH Terms] OR "Lao PDR"[All Fields] OR "Sri Lanka"[MeSH Terms] OR "Cape Verde"[All Fields] OR "lesotho"[MeSH Terms] OR "tanzania"[MeSH Terms] OR "cambodia"[MeSH Terms] OR "mauritania"[MeSH Terms] OR "Timor-Leste"[MeSH Terms] OR "cameroon"[MeSH Terms] OR "micronesia"[MeSH Terms] OR "tunisia"[MeSH Terms] OR "comoros"[MeSH Terms] OR "moldova"[MeSH Terms] OR "ukraine"[MeSH Terms] OR "Congo Republic"[All Fields] OR "mongolia"[MeSH Terms] OR "uzbekistan"[MeSH Terms] OR "Cote d'Ivoire"[MeSH Terms] OR "morocco"[MeSH Terms] OR "vanuatu"[MeSH Terms] OR "djibouti"[MeSH Terms] OR "myanmar"[MeSH Terms] OR "vietnam"[MeSH Terms] OR "egypt"[MeSH Terms] OR "nepal"[MeSH Terms] OR "West Bank and Gaza"[All Fields] OR "El Salvador"[MeSH Terms] OR "nicaragua"[MeSH Terms] OR "zambia"[MeSH Terms] OR "eswatini"[MeSH Terms] OR "nigeria"[MeSH Terms] OR "zimbabwe"[MeSH Terms] OR "ghana"[MeSH Terms] OR "pakistan"[MeSH Terms] OR "albania"[MeSH Terms] OR "American Samoa"[MeSH Terms] OR "argentina"[MeSH Terms] OR "armenia"[MeSH Terms] OR "azerbaijan"[MeSH Terms] OR "republic of belarus"[MeSH Terms] OR "belize"[MeSH Terms] OR "Bosnia and Herzegovina"[MeSH Terms] OR "botswana"[MeSH Terms] OR "brazil"[MeSH Terms] OR "bulgaria"[MeSH Terms] OR "china"[MeSH Terms] OR "colombia"[MeSH Terms] OR "Costa Rica"[MeSH Terms] OR "cuba"[MeSH Terms] OR "dominica"[MeSH Terms] OR "Dominican Republic"[MeSH Terms] OR "Equatorial Guinea"[MeSH Terms] OR "ecuador"[MeSH Terms] OR "fiji"[MeSH Terms] OR "gabon"[MeSH Terms] OR ("georgia"[MeSH Terms] OR "georgia republic"[MeSH Terms]) OR "grenada"[MeSH Terms] OR "guatemala"[MeSH Terms] OR "guyana"[MeSH Terms] OR "indonesia"[MeSH Terms] OR "iran"[MeSH Terms] OR "iraq"[MeSH Terms] OR "jamaica"[MeSH Terms] OR "jordan"[MeSH Terms] OR "kazakhstan"[MeSH Terms] OR "kosovo"[MeSH Terms] OR "lebanon"[MeSH Terms] OR "libya"[MeSH Terms] OR "malaysia"[MeSH Terms] OR "mexico"[MeSH Terms] OR "montenegro"[MeSH Terms] OR "namibia"[MeSH Terms] OR "paraguay"[MeSH Terms] OR "peru"[MeSH Terms] OR "samoa"[MeSH Terms] OR "serbia"[MeSH Terms] OR "South Africa"[MeSH Terms] OR "suriname"[MeSH Terms] OR "thailand"[MeSH Terms] OR "tonga"[MeSH Terms] OR "turkey"[MeSH Terms] OR "turkmenistan"[MeSH Terms] OR "micronesia"[MeSH Terms] OR "venezuela"[MeSH Terms]) | 5,417,942 |
| 16 | "Dynami*" [All Fields] OR "dynami*" [MeSH Terms] OR "Utilizatio*" [All Fields] OR "utilizatio*" [MeSH Terms] OR "use" [All Fields] OR "practic*" [MeSH Terms] OR "practic*" [All Fields] OR "unmet nee*" [All Fields] OR "discontinuatio*"[All Fields] OR "dis-continuatio*" [All Fields] OR "switchin*" [All Fields] OR "chang*" [All Fields] OR "chang*" [MeSH Terms] |  |  | "dynami*"[All Fields] OR "dynami*"[MeSH Terms] OR "utilizatio*"[All Fields] OR "utilizatio*"[MeSH Terms] OR "use"[All Fields] OR "practic*"[MeSH Terms] OR "practic*"[All Fields] OR "unmet nee*"[All Fields] OR "discontinuatio*"[All Fields] OR "dis continuatio*"[All Fields] OR "switchin*"[All Fields] OR "chang*"[All Fields] OR "chang*"[MeSH Terms] | 8,983,012 |
| 15 | "Birth control" [All Fields] OR "family planning services" [All Fields] OR "family planning services" [MeSH Terms] OR "contraception behavior" [MeSH Terms] OR "contraception/psychology" [MeSH Terms] OR "contraception/utilization" [All Fields] OR "family plannin*" [All Fields] OR "contracepti*" [All Fields] OR "contracepti*" [MeSH Terms] OR "contraceptive agen*" [All Fields] OR "contraceptive agen*" [MeSH Terms] OR "contraceptive methods" [All Fields] OR "contraceptive device*" [MeSH Terms] OR "contraceptive devic*" [All Fields] OR "planned pregnanc*" [All Fields] OR "birth prevention*" [All Fields] OR "prevent pregnanc*" [All Fields] OR "birth interva*" [MeSH Terms] OR "birth interva**" [All Fields] OR "birth spacing" [All Fields] OR "pregnancy interval" [All Fields] OR "pregnancy spacing" [All Fields] |  |  | "Birth control"[All Fields] OR "family planning services"[All Fields] OR "family planning services"[MeSH Terms] OR "contraception behavior"[MeSH Terms] OR "contraception/psychology"[MeSH Terms] OR "contraception/utilization"[All Fields] OR "family plannin*"[All Fields] OR "contracepti*"[All Fields] OR "contracepti*"[MeSH Terms] OR "contraceptive agen*"[All Fields] OR "contraceptive agen*"[MeSH Terms] OR "contraceptive methods"[All Fields] OR "contraceptive device*"[MeSH Terms] OR "contraceptive devic*"[All Fields] OR "planned pregnanc*"[All Fields] OR "birth prevention*"[All Fields] OR "prevent pregnanc*"[All Fields] OR "birth interva*"[MeSH Terms] OR "birth interva*"[All Fields] OR "birth spacing"[All Fields] OR "pregnancy interval"[All Fields] OR "pregnancy spacing"[All Fields] | 189,987 |
| 14 | "Disabili*" [All Fields] OR "disabili*" [MeSH Terms] OR "impairmen*" [All Fields] OR "physical disabilit*" [All Fields] OR "visual impairmen*"[All Fields] OR "visual loss" [All Fields] OR "blind" [All Fields] OR blind [MeSH Terms] OR "hearing loss"[All Fields] OR "hearing loss" [MeSH Terms] OR "hearing impairmen*" [All Fields] OR "deaf" [All Fields] OR "intellectual disabili*" [All Fields] OR "intellectual disabili*" [MeSH Terms] OR "sensory disabili*" [All fields] |  |  | "disabili*"[All Fields] OR "disabili*"[MeSH Terms] OR "impairmen*"[All Fields] OR "physical disabilit*"[All Fields] OR "visual impairmen*"[All Fields] OR "visual loss"[All Fields] OR "blind"[All Fields] OR "visually impaired persons"[MeSH Terms] OR "blindness"[MeSH Terms] OR "hearing loss"[All Fields] OR "hearing loss"[MeSH Terms] OR "hearing impairmen*"[All Fields] OR "deaf"[All Fields] OR "intellectual disabili*"[All Fields] OR "intellectual disabili*"[MeSH Terms] OR "sensory disabili*"[All Fields] | 1,081,095 |
| 13 | "women" [All Fields] OR "women" [MeSH Terms] OR "reproductive age" [All Fields] OR "15-49 years" [All Fields] |  |  | "women"[All Fields] OR "women"[MeSH Terms] OR "reproductive age"[All Fields] OR "15-49 years"[All Fields] | 1,364,007 |

**Search results from Pub Med**
